# Draft Genome Sequences of *Edwardsiella ictaluri* Strains LADL11-100 and LADL11-194 Isolated from Zebrafish *Danio rerio*

**DOI:** 10.1128/genomeA.01449-15

**Published:** 2015-12-17

**Authors:** Rui Wang, Hasan C. Tekedar, Mark L. Lawrence, Vladimir N. Chouljenko, Joohyun Kim, Nayong Kim, Konstantin G. Kousoulas, John P. Hawke

**Affiliations:** a Department of Pathobiological Sciences, School of Veterinary Medicine, Louisiana State University, Baton Rouge, Louisiana, USA; b School of Veterinary Medicine, Division of Biotechnology and Molecular Medicine, Louisiana State University, Baton Rouge, Louisiana, USA; c Department of Basic Sciences, College of Veterinary Medicine, Mississippi State University, Mississippi, USA; d Center for Computation and Technology, Louisiana State University, Baton Rouge, Louisiana, USA

## Abstract

Here, we report the draft genome sequences of *Edwardsiella ictaluri* strains LADL11-100 and LADL11-194, two isolates from natural outbreaks of edwardsiellosis in the zebrafish *Danio rerio*, as well as the sequences of the plasmids carried by the zebrafish strain of *E. ictaluri.*

## GENOME ANNOUNCEMENT

*Edwardsiella ictaluri*, the causative agent of enteric septicemia of channel catfish (ESC), was first described in channel catfish in 1979, and the genome of a typical catfish strain, LADL93-146, was released in 2012 (1–3). Zebrafish can be used as an experimental model for edwardsiellosis by immersion and injection using the catfish strain (4). However, it was not until 2013 that the zebrafish *Danio rerio* was reported as a natural host of *E. ictaluri* infection (5). Specimens from Louisiana State University, Baton Rouge, LA, and the University of Massachusetts at Amherst, Amherst, MA, sent to the Louisiana Aquatic Diagnostic Laboratory (LADL), Baton Rouge, LA, for diagnosis were found to be infected with *E. ictaluri*. Zebrafish strains LADL11-100 and LADL11-194 were further identified as the primary causes of high mortality in both facilities. These strains are considered typical of other zebrafish strains isolated from other laboratories and geographic locations but vary consistently from the catfish strains in many aspects (3).

To investigate the potential genetic variation and virulence mechanisms of zebrafish strains in relation to catfish strains, *E. ictaluri* LADL11-100 and LADL11-194 genomic DNA sequencing was performed using the Ion Torrent Personal Genome Machine (PGM) and 316 sequencing Chip (Life Technologies) at Louisiana State University, Division of Biotechnology and Molecular Medicine (BioMMED). *E. ictaluri* was grown in brain heart infusion (BHI) broth for 18 h, and the genomic DNA was extracted using the High Pure PCR template preparation kit (Roche Applied Science). The Ion Xpress Plus fragment library kit (Life Technologies) was used to prepare high-quality fragment libraries. Template-positive Ion Sphere Particles (ISPs) containing clonally amplified DNA were prepared for sequencing using the Ion PGM 200 sequencing kit.

The sequencing of the two strains produced output totals of 4,020,880 and 2,894,144 usable reads, with average read lengths of 221 bp and 217 bp. The average G+C content of both zebrafish strains is 57.4%, which is identical to that of catfish strain *E. ictaluri* LADL93-146. CLC Genomics Workbench version 7.5 (CLC bio) and Sequencher 5.2.4 (Gene Codes Corporation) used for assembly yielded 127 and 130 contigs that were >1,000 bp for LADL11-100 and LADL11-194, respectively. The maximum length of the contigs from each strain is approximately 160 kb.

The genome was annotated using the RAST server (6, 7) and NCBI Prokaryotic Genome Annotation Pipeline (PGAP) (http://www.ncbi.nlm.nih.gov/genome/annotation_prok/). RAST predicted a total of 3,613 coding DNA sequences (CDS) and 85 RNAs for LADL11-100 and 3,638 CDSs and 94 RNAs for LADL11-194. These draft genome sequences, together with that of *E. ictaluri* LADL93-146 (accession no. CP001600), were uploaded to CONTIGuator for a comparative analysis (8). Following analysis of both zebrafish strains, 111 contigs of the strains were aligned to the catfish strain of *E. ictaluri.*

### Nucleotide sequence accession numbers.

The complete genome sequences of two zebrafish strains have been deposited at DDBJ/EMBL/GenBank under accession numbers LDWX00000000 (LADL11-100 chromosome) and LEAL00000000 (LADL11-194 chromosome). The plasmids in the zebrafish strain LADL11-100 (5), named pEIZ1 and pEIZ2, were sequenced as well, and the accession numbers for these plasmids are KR869777 (plasmid pEIZ1) and KR869778 (plasmid pEIZ2).

## References

[1] Hawke JP 1979 A bacterium associated with disease of pond cultured channel catfish, *Ictalurus punctatus*. J Fish Res Board Can 36:1508–1512. doi:10.1139/f79-219.

[2] Hawke JP, McWhorter AC, Steigerwalt AG, Brenner DJ 1981 *Edwardsiella ictaluri* sp. nov., the causative agent of enteric septicemia of catfish. Int J Syst Bacteriol 31:396–400. doi:10.1099/00207713-31-4-396.

[3] Williams ML, Gillaspy AF, Dyer DW, Thune RL, Waldbieser GC, Schuster SC, Gipson J, Zaitshik J, Landry C, Banes MM, Lawrence ML 2012 Genome sequence of *Edwardsiella ictaluri* 93-146, a strain associated with a natural channel catfish outbreak of enteric septicemia of catfish. J Bacteriol 194:740–741. doi:10.1128/JB.06522-11. 22247535 PMC3264089

[4] Petrie-Hanson L, Romano CL, Mackey RB, Khosravi P, Hohn CM, Boyle CR 2007 Evaluation of zebrafish *Danio rerio* as a model for enteric septicemia of catfish (ESC). J Aquat Anim Health 19:151–158. doi:10.1577/H06-026.1. 18201056

[5] Hawke JP, Kent M, Rogge M, Baumgartner W, Wiles J, Shelley J, Savolainen LC, Wagner R, Murray K, Peterson TS 2013 Edwardsiellosis caused by *Edwardsiella ictaluri* in laboratory populations of zebrafish *Danio rerio*. J Aquat Anim Health 25:171–183. doi:10.1080/08997659.2013.782226. 23865817 PMC4077847

[6] Overbeek R, Olson R, Pusch GD, Olsen GJ, Davis JJ, Disz T, Edwards RA, Gerdes S, Parrello B, Shukla M, Vonstein V, Wattam AR, Xia F, Stevens R 2014 The SEED and the rapid annotation of microbial genomes using subsystems technology (RAST). Nucleic Acids Res 42:D206–D214. doi:10.1093/nar/gkt1226. 24293654 PMC3965101

[7] Aziz RK, Bartels D, Best AA, DeJongh M, Disz T, Edwards RA, Formsma K, Gerdes S, Glass EM, Kubal M, Meyer F, Olsen GJ, Olson R, Osterman AL, Overbeek RA, McNeil LK, Paarmann D, Paczian T, Parrello B, Pusch GD, Reich C, Stevens R, Vassieva O, Vonstein V, Wilke A, Zagnitko O 2008 The RAST server: Rapid Annotations using Subsystems Technology. BMC Genomics 9:75. doi:10.1186/1471-2164-9-75. 18261238 PMC2265698

[8] Galardini M, Biondi EG, Bazzicalupo M, Mengoni A 2011 CONTIGuator: a bacterial genomes finishing tool for structural insights on draft genomes. Source Code Biol Med 6:11. doi:10.1186/1751-0473-6-11. 21693004 PMC3133546

